# Generational differences in the relationship between media exposure and health behaviors during COVID-19 pandemic

**DOI:** 10.3389/fpsyg.2023.1039122

**Published:** 2023-02-16

**Authors:** Ruimin He, Jia He, Huan Zhang

**Affiliations:** ^1^School of Media and Communication, Shanghai Jiao Tong University, Shanghai, China; ^2^USC-SJTU Institute of Cultural and Creative Industry, Shanghai Jiao Tong University, Shanghai, China; ^3^School of Journalism and Communication, Nankai University, Tianjin, China

**Keywords:** media exposure, social cognitive theory, protection motivation theory, health behaviors, generational differences

## Abstract

Based on a questionnaire survey (*N* = 857), this study analyzed generational differences in the public health behaviors of COVID-19 and provided an explanation for generational differences from the perspective of media exposure. There are significant differences in media exposure and health behaviors between the Mesozoic generation (35–55) and the young generation (18–34) during the lull. The Mesozoic generation paid greater attention to information on pandemics. Consequently, their health behaviors surpass that of the young generation. On the basis of social cognitive theory and protection motivation theory, this study develops a mediating model of media exposure on health behaviors, demonstrating that media exposure can influence health behaviors through the mediating effects of perceived severity, self-efficacy, and response efficacy, but not *via* perceived susceptibility. Moreover, a moderated mediation study found that generation moderates the indirect effect of media exposure on health behaviors *via* perceived susceptibility. Media exposure influences Mesozoic healthy behaviors positively by decreasing their perceived susceptibility. The implication of this study is that the development of health communication theory must account for generational differences and disease-specific characteristics.

## 1. Introduction

The relentless invasion of the COVID-19 pandemic has drastically altered the spring of 2020, and China was the first nation to be affected. It is the most critical public health emergency since the formation of New China, with the quickest spread, the broadest infectious spectrum, and the most challenging prevention and management (Xinhuanet., 2020). A public health emergency is a situation in which a health threat causes an imminent risk or serious harm to a large population (Haffajee et al., 2014). The public receives a great deal of pertinent information, instructing them to wear masks, take temperature, wash their hands and disinfect more often, stay indoors, etc. Existing research on SARS and MERS has demonstrated that different demographic variables are associated with distinct prevention behaviors in infectious diseases (Brug et al., 2004; Glanz and Bishop, 2010).

At the start of the COVID-19 outbreak, however, “how to persuade parents to wear masks” topped the trending search terms on major social media platforms such like Weibo. Under this issue, many young people complained that their parents paid little attention to COVID-19 and related similar experiences. Some individuals remarked, “If it were not for this pandemic, we might not realize how challenging it is to connect with our parents. They still go out without wearing masks and even mock us for making a fuss.” In addition, research has indicated that during an outbreak of COVID-19, young people are more prone to adopt severe preventive measures and are more likely to express their thoughts on the Internet (Chu et al., 2020). Generational differences in health behaviors are prominent, which some scholars have called “intergenerational battles” (Tang, 2020). why are there generational differences in health behaviors? What are the characteristics and reasons for generational differences? These are the main issues that the study wants to explore.

When public health emergencies arise, people seek information through many channels, with media coverage of the events as the most crucial source (Holland et al., 2012). The Measurement of how people are “exposed” to media content is essential to understanding media use and its effects (De Vreese and Neijens, 2016). Media exposure influences people’s perception of the threat, which may ultimately determine their response to the crisis (Coleman and Thorson, 2002). Several studies have shown generational differences in media exposure (Xue et al., 2018).

The literature on media exposure and health behaviors focuses primarily on nonurgent risks, with few studies examining protective actions during a public health emergency (Slater et al., 2007; Siu, 2008). the media exposure of the public during public health emergencies was ignored. Consequently, our study attempts to fill this gap. In addition, media exposure encompasses the audience’s contact frequency and substance. However, most relevant studies (Coleman, 1993; Dougall et al., 2005) only examined the frequency of exposure. Therefore, this study will analyze the effects of media exposure from the perspectives of exposure frequency and exposure extensity, which refers to the amount of information the audience is exposed to, enabling them to get a more comprehensive understanding of pandemics (Li, 2018). Besides, the majority of studies mainly relied on legacy media while excluding interpersonal communication (Zhang et al., 2020). This investigation will focus on media exposure at the mass, group organization, and interpersonal levels.

Dutta and Zoller (2009) classify health communication research as post-positivistic, interpretative, critical, and cultural. Based on the tradition of social psychology, the post-positivistic approach emphasizes the analysis of communication and social and psychological variables to explain and predict health behaviors (Dutta and Zoller, 2009; Thompson, 2011). This approach holds a prominent position in the field of health communication research. Under the post-positivistic approach, health communication extensively uses behavioral science theories, such as social cognitive theory and protection motivation theory. And these two theories focus on the effects of communication on the four Social-Cognitive variables of perceived severity, perceived susceptibility, self-efficacy, and response efficacy.

In summary, this research concludes by examining generational differences in health behaviors concerning media exposure and individual psychological and cognitive aspects. The conclusion describes the characteristics of generational differences in health behaviors as well as the mechanisms that provide an impact. It can further illuminate theories on the influence mechanisms between media exposure and health behaviors and improve future health communication effects.

## 2. Literature review

### 2.1. Social-cognitive predictors of health behaviors

Various theories of health behavior, including social cognitive theory, protective motivation theory, the extended parallel process model, the health belief model, and the theory of planned behavior, all suggest that socio-cognitive psychology substantially affects health behavior.

Bandura (1986) proposed, from the standpoint of individual cognition, the social cognitive theory, which held that health behaviors are influenced by two cognitive variables: self-efficacy and outcome expectations. Self-efficacy, a central notion of social cognitive theory, relates to a person’s perception of their capacity to engage in essential health activities (Bandura, 1997). This idea proposes that strengthening an individual’s self-efficacy can effectively enhance health practices (Bandura, 2004). Schunk and Usher (2019) suggest that cognitive elements should incorporate beliefs, perceptions, and emotions. However, previous empirical research has frequently just examined self-efficacy and outcome expectations (Boateng et al., 2016). According to Glanz and Bishop (2010), the social cognitive theory is the most often utilized theory in health behavior research.

Protection motivation theory proposes, based on social cognitive theory, that the intention to conduct a protective behavior is determined by two concurrent cognitive and partially emotional processes: one is threat appraisal, which refers to an individual’s evaluation of the potential risk. The threat appraisal consisted of two variables: perceived severity and perceived susceptibility. The coping appraisal refers to an individual’s evaluation of his or her ability to deal with danger and typically consists of the variables self-efficacy and response efficacy (Milne et al., 2000). According to the hypothesis, perceived susceptibility, perceived severity, response efficacy, and self-efficacy might favorably influence health behavior (Prentice-Dunn and Rogers, 1986). In addition, empirical research has demonstrated that the protection motivation theory is frequently applied to preventive health behaviors such as physical activity, cancer screening, and substance addiction and possesses superior predictive value (Witte, 1994; Roberto et al., 2007; Gaston and Prapavessis, 2014).

Regarding risk perception and health behaviors, perceived severity refers to the assumption that the consequences of contracting the disease are severe for the individual and others. And perceived susceptibility is a person’s perception of their likelihood of experiencing a risk or contracting an ailment or sickness (Tanner et al., 1991). Previous research has demonstrated that both factors significantly influence health behavior adoption (Kasmaei et al., 2014). Regarding efficacy and health practices, Since Bandura (1986) established self-efficacy, numerous empirical researches have demonstrated that self-efficacy positively predicts preventative disease practices (McCann et al., 1995). Response efficacy is related to self-efficacy and refers to an individual’s conviction in the success of steps to lower health risks (Witte, 1994). The greater a person’s perception of a preventive measure’s efficacy, the more likely they are to adopt the practice (Floyd et al., 2000). Numerous studies have demonstrated that response efficacy increases the propensity to protect oneself and others (Lwin et al., 2010).

The study, therefore, focuses primarily on the public’s preventive health activities during the COVID-19 pandemic and suggests the following hypothesis:

*H1*: Perceived susceptibility affects health behaviors positively.

*H2*: Perceived severity affects health behaviors positively.

*H3*: Self-efficacy affects health behaviors positively.

*H4*: response efficacy affects health behaviors positively.

### 2.2. Media exposure and health behavior

The social cognitive theory proposed a “triadic interaction model” including personal factors, behavior, and the environment (Bandura, 1998). The consideration of environmental factors in social cognitive theory includes both the physical and social environment (Casper, 2001). Additionally, someone pointed out that the protection motivation theory focuses primarily on the influence of personal factors on health behavior and disregards environmental elements (Lebek et al., 2014). Media exposure is a critical socio-environmental factor (Narayan, 2013). The media significantly alter public health perceptions and encourage health behavior (Mullins et al., 2008). Studies demonstrate that mass media health messages can have favorable behavioral impacts (Wakefield et al., 2010). Through meta-analysis, Laranjo et al. (2015) determined that social media use can encourage individual behavior change. Consequently, this study integrates social cognitive theory and protection motivation theory to examine how media exposure influences health behavior by working on intrinsic cognitive mechanisms.

On the one hand, the media is the primary source of risk perception (Keown, 1989). Researchers have discovered that exposure to the news media influences the impression of influenza H1N1 risk (Lin and Lagoe, 2013; Oh et al., 2015). Other research has demonstrated that exposure to health-related news influences people’s perceptions of health threats and induces behavioral responses (Wei et al., 2008). In addition, El-Toukhy (2015) found that social media exposure to disease information altered the perception of disease severity and susceptibility. Exposure to COVID-19 material from both mass media and social media enhances perceived severity, perceived susceptibility, and COVID-19 preventative behaviors (Ranjit et al., 2021; Truong et al., 2022).

However, on the other hand, Bandura (1977) believes four key sources produce self-efficacy: mastery experiences, vicarious experiences, verbal persuasion, and physiological and affective states. Vicarious experiences and verbal persuasion highlight the impact of external factors on self-efficacy. Undoubtedly, the media may contribute to disseminating vicarious experiences and implementing verbal persuasion (Gibbons et al., 2010). Several studies have demonstrated that media exposure to health-related information can increase an individual’s self-efficacy. For instance, Bass et al. (2006) discovered a statistically significant positive association between Internet usage and self-efficacy. Two months of exposure to health information resulted in a considerable rise in self-efficacy among cancer patients, according to Lee et al. (2008). Response efficacy, which relates to the effectiveness of protective behaviors, is also influenced by external factors, including vicarious experience and verbal persuasion. Therefore, it is plausible to hypothesize that media exposure is also an external factor influencing response efficacy. In addition, a study confirms that during the COVID-19 pandemic, media exposure can indirectly influence public health behaviors *via* the mediation effects of self-efficacy and response efficacy (Ma, 2022).

The paper primarily proposes the following hypotheses:

*H5*: media exposure influences health behaviors positively through the mediation of perceived susceptibility.

*H6*: media exposure influences health behaviors positively via the moderating effect of perceived severity.

*H7*: media exposure influences health behaviors positively via the mediating effect of response efficacy.

*H8*: media exposure influences health behaviors positively through the mediation of self-efficacy.

### 2.3. Generational differences

A generation is a recognizable group that shares birth years, age range, and critical life experiences throughout crucial developmental phases (Kupperschmidt, 2000). Due to their similar location, people share comparable experiences and hence generate similar thoughts, experiences, and behavior patterns (Mannheim, 2002). Additionally, these unique life experiences distinguish one generation from the next (Jurkiewicz and Brown, 1998). Consequently, generational differences refer to the differences in cognition, attitude, and behavior choice across various generations.

According to research in the field of communication, there are discernible generational differences in media exposure. Regarding media types, the old choose newspapers and television to receive information (Lauf, 2001), while the young prefer the Internet and other electronic media (Pierce et al., 1990). In terms of media content, a study (Dou et al., 2006) reveals that the Chinese × generation pays more attention to television series and other amusement programs and less attention to economic news and other information programs than prior generations.

In addition, scholars consider the consequences of media exposure on various generations’ cognition, attitudes, and actions. Putnam (2000) discovered that young heavy Internet users in the United States were more separated from public life, less engaged in social activities, and less trustworthy of their peers. Some research, focusing on health behaviors has shown that the digital divide created by generational differences has a significant impact on people’s health levels (Viswanath and Kreuter, 2007; Hong et al., 2017). Moreover, researchers have demonstrated that the influence of media on risk perception varies between populations (Sussman et al., 1989; Snyder and Rouse, 1995). According to Zhou and Yang (2020), the generational gap in the adoption and use of digital media influences the generational gap in “knowing, believing, and doing” regarding health. However, previous research on health behavior mostly tended to consider respondents as a whole, neglecting the diverse effects on distinct populations.

**Figure 1:**
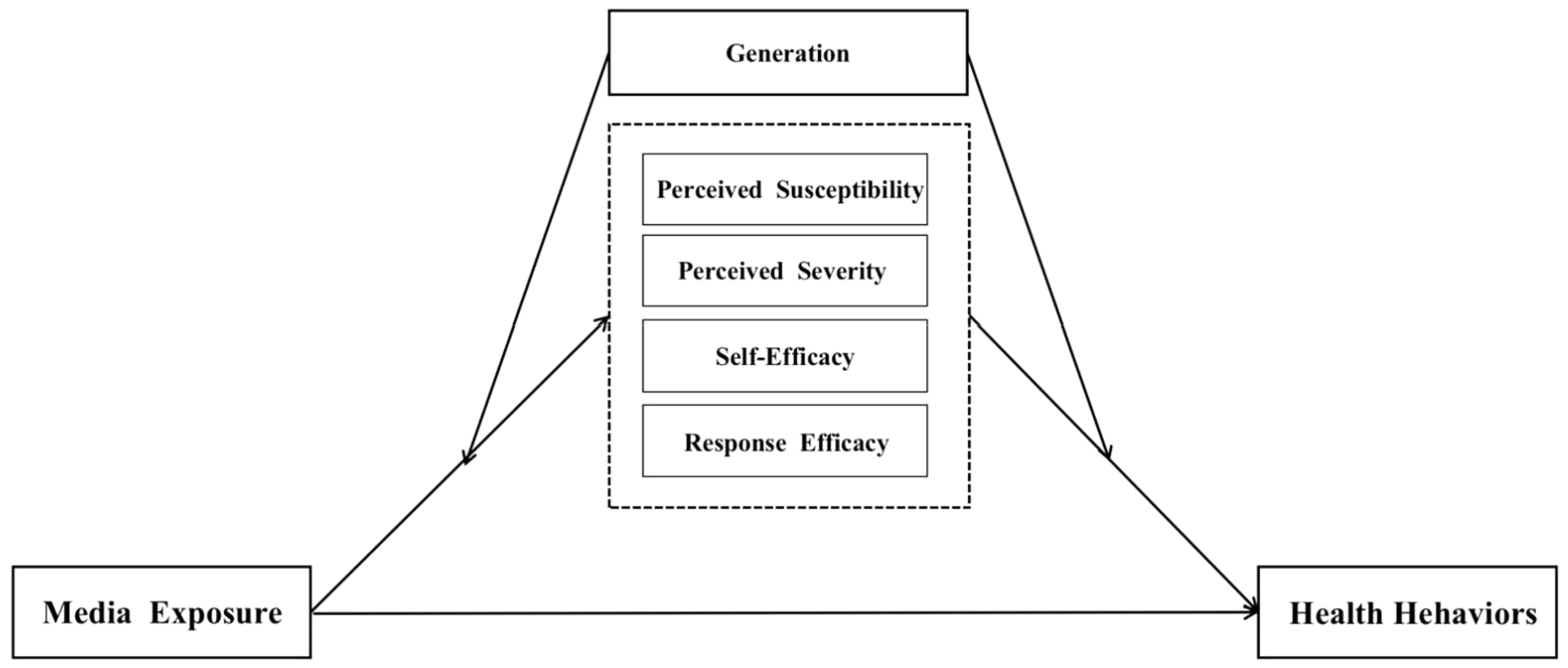
The Moderated mediation model to test the influence of generation on the mediated relationship between media exposure and health behaviors.

Therefore, it is impossible to generalize whether media exposure to risk information about the COVID-19 pandemic influences relevant preventive health behaviors across generations. Consequently, this study expects that the mediating role of the aforementioned intrinsic cognitive factors in media exposure and health behaviors varies between generations as shown in Figure 1, and asks the following research questions:

RQ1: Are there significant generational differences in COVID-19 media exposure?
RQ2: Are there significant generational differences in COVID-19-preventive health behaviors?
RQ3: Are the mediating roles of intrinsic cognitive factors (perceived severity, perceived susceptibility, self-efficacy, response efficacy) in media exposure and health behaviors moderated varies between generations?

## 3. Method

### 3.1. Participants and procedures

From June 28 to July 4, 2020, we conducted a survey using the Tencent questionnaire platform^1^ to evaluate the hypotheses. The total sample consisted of 857 Chinese citizens. In the pre-test phase, ambiguous and difficult-to-understand questions were altered based on audience comments. In addition, it is important to note that people’s psychological cognition and health behaviors fluctuate at various pandemic stages. When the questionnaire was issued, the pandemic in China had entered a lull. A June outbreak in Beijing raised anxiety, but the situation is again under control. Wenhong Zhang stated in an interview on July 9 that, based on the epidemiological history, the occasional confirmed case is typical and does not result in a rapid spread.

In terms of the procedures, First, an independent sample t-test was adopted for analyzing the health behaviors and media exposure of the young and Mesozoic generations. We then test the association between variables using multiple regression analysis. In addition, to verify the hypotheses proposed, we built a moderated mediation model. an indirect effects analysis was performed with Hayes’s (2009) method, testing standard errors *via* bootstrapping using Preacher and Hayes’s SPSS macro. And finally, the moderating effect of generations, for both paths of mediating effects, is examined utilizing model 58 in PROCESS.

The general characteristics of the study participants are shown in Table 1. Males made up 47.3% of the sample (405) while females made up 52.7% (452). The majority (73.3%) lived in urban regions, with 71 (8.3%) in junior high school or lower, 149 (17.4%) in high school and technical secondary school, 232 (27.1%) in college, 39.3% (337) having earned a university degree, and others with master’s and doctoral degrees. Participants were questioned about any recently confirmed instances in their home. 28% of respondents indicated yes.

**Table 1:** Descriptive statistical analysis of samples.

Category	Variable	*N* (%)	
Gender	Male	405	47.3
	Female	452	52.7
	Total	857	100
Education level	Junior high school or lower	71	8.3
	High school, technical secondary school	149	17.4
	College	232	27.1
	Undergraduate	337	39.3
	Postgraduate	68	7.9
	Total	857	100
Residence	Urban	628	73.3
	Rural	229	26.7
	Total	857	100
Exist any newly confirmed cases in your region?	Yes	247	28.8
	No	610	71.2
	Total	857	100

### 3.2. Measurements

Media exposure: frequency of access to news about the COVID-19 pandemic and extensity of news messages accessed from the COVID-19 pandemic (Li, 2018).

In accordance with Li (2018), the extensity of media exposure during a pandemic was determined by how comprehensively one accessed the following news messages: (a) international pandemic situation; (b) domestic pandemic situation; (c) the severe impact of the pandemic (e.g., on individuals, regions, and countries); (d) government prevention and control measures; and (e) knowledge of pandemic prevention and popularization (*α* = 0.819).

The frequency of media exposure was measured by the number of times individuals accessed news about the COVID-19 pandemic *via* the following media channels: (a) Channels of mass communication; (b) Interpersonal channels; (c) Organizational channels; and (d) Social media channels (*α* = 0.734). The frequency and extensity of media exposure were measured using a 5-point Likert scale (1 = never, 5 = frequently). After calculating the scale’s weight by principal component analysis, the total score was then determined.

Threat appraisal and Coping appraisal: Refer to the scale created and validated by Witte (1994) and use principal component analysis to derive factors for 12 items. There was a total of four components, the KMO value was 0.763, Bartlett’s sphericity test level was 0.000, and the explainable variance was 70.75%. The four factors are as follows: perceived susceptibility, a total of three items, including “I am at risk of contracting Covid-19” (*α* = 0.897); perceived severity, a total of three items, including “I believe once I have Covid-19 it may be life-threatening” (*α* = 0.709); self-efficacy, a total of two items, including “I think my physical fitness can resist the virus” (*α* = 0.674); response efficacy, a total four items in all, including “I believe wearing masks and engaging in other preventive actions can effectively contain the pandemic” (*α* = 0.788).

Health behaviors: During the pandemic, the media promotes preventative health behaviors including wearing masks, frequent hand washing, frequent disinfection, and avoiding gatherings. After the incident, some experts reiterated the significance of using public chopsticks or serving chopsticks. In addition, the media widely publicized the advantages of using public chopsticks. Respondents scored their self-assessment on preventive behavior measures, such as wearing masks, washing hands, disinfecting furnishings or workspaces, avoiding gathering activities, and utilizing public chopsticks (1–5 points for each item, *α* = 0.819).

Generation is the central variable in our investigation. The division of generations occurs in various ways. This study refers fully to Xue et al.’s (2018) practice of dividing generations based on variations in media use and the practice of separating generations based on differences in preventive behaviors (Kim and Crimmins, 2020), dividing the interviewees into two generations: the young generation, inhabitants under the age of 34, and the Mesozoic generation, residents between the ages of 35 and 55. Due to the limits of online questionnaires, it is not possible to collect data on a large number of inhabitants beyond the age of 55. Hence this study focuses on the young generation (18–34) and Mesozoic generation (35–55).

## 4. Results

### 4.1. Descriptive statistics and variance analysis

Table 2 reveals that the content of media exposure (*M* = 4.16; SD = 0.68) is extensive and the frequency of media exposure is high, indicating that people continue to pay close attention to the COVID-19 pandemic during the calm phase. In addition, public health behaviors have a high score (*M* = 4.11; SD = 0.80), which demonstrates their voluntary adherence to healthy behavior norms. Moreover, response efficacy (*M* = 4.25; SD = 0.73) was the highest perception of the pandemic, followed by perceived severity (*M* = 3.62; SD = 0.97), self-efficacy (*M* = 3.33; SD = 0.97) and perceived susceptibility (*M* = 1.84; SD = 1.01). With the pandemic effectively under control, the number of confirmed cases in all but a few regions have been reduced to zero. Therefore, the perceived susceptibility of individuals is not high. And The analysis of correlation between variables also gives conditions for further regression and mediation analyses. According to Table 2, in addition to perceived susceptibility, perceived severity, self-efficacy, and response efficacy are positively related to the frequency (*r* = 0.18; *r* = 0.44; *r* = 0.36) and the extensity (*r* = 0.27; *r* = 0.20; *r* = 0.42) of media exposure and health behaviors (*r* = 0.23; *r* = 0.23; *r* = 0.47). However, there is a negative correlation between perceived susceptibility and self-efficacy (*r* = −0.07), response efficacy (*r* = −0.07), which will be discussed further in the subsequent analysis.

**Table 2:** Mean value, standard deviation and correlation coefficient of each variable.

Variables	*M*	SD	1	2	3	4	5	6	7	8	9	10	11
1. Frequency	3.88	0.74	/	0.56^**^	0.30^**^	−0.17	0.18^**^	0.18^**^	0.36^**^	0.12	0.44	−0.52	−0.03
2. Extensity	4.16	0.68		/	0.41^**^	0.03	0.27^**^	0.20^**^	0.42^**^	0.66	0.38	−0.14^**^	−0.04
3. Behaviors	4.11	0.80			/	−0.06	0.23^**^	0.23^**^	0.47^**^	0.95^**^	−0.04	−0.05	−0.03
4. Susceptibility	1.84	1.01				/	0.31^**^	−0.07^*^	−0.07^*^	−0.07^*^	−0.05	−0.06	−0.74^*^
5. Severity	3.62	0.97					/	−0.05	0.32^**^	−0.01	−0.08^*^	−0.05	−0.05
6. Self-efficacy	3.33	0.97						/	0.28^**^	0.06	0.04	−0.11^**^	−0.02
7. Response efficacy	4.25	0.73							/	0.02	0.05	−0.10^**^	0.02
8. Gender	/	/								/	−0.04	−0.04	−0.02
9. Education level	/	/									/	−0.21^**^	−0.19^**^
10. Residence	/	/										/	0.15^**^
11. Regional risk	/	/											/

^*^p < 0.05, ^**^p < 0.01.

The independent sample *t*-tests were then used to assess the generational differences in media exposure and health behaviors between the young and Mesozoic generations. Regarding media exposure frequency, there were no discernible changes between the two generations. Specifically, as indicated in Table 3, there is a significant difference between the Mesozoic and young generations in terms of interpersonal media exposure channels (*t* (857) = −1.97, *p* = 0.05). Mesozoic exposure frequency on interpersonal channels (*M* = 3.66; SD = 1.05) is substantially greater than that of young individuals (*M* = 3.53; SD = 0.97). The average difference in score is −0.138 (d = −0.13), 95%CI = [−0.275.0.000]. Moreover, there is a significant difference between these two generations on organizational channels (*t* (857) = −3.22, *p* = 0.001). The frequency of Mesozoic exposure to organizational channels (*M* = 3.62; SD = 1.12) is substantially more than that of the young (*M* = 3.37; SD = 1.11). The average difference in score is −0.249 (*d* = −0.22), 95 percent CI = [−0.401, –0.097]. And there is a substantial difference between the two generations in terms of media exposure intensity (*t* (857) = −4.14, *p* < 0.001). The score of the Mesozoic public (*M* = 4.27; SD = 0.68) is higher than that of the young (*M* = 4.08; SD = 0.67), The average score difference is −0.19 (*d* = −0.28). 95%CI = [−0.287, –0.102]. During the pandemic pause, the Mesozoic generation was substantially more interested in pandemic-related news than the young generation.

**Table 3:** An analysis of differences in media exposure between generations.

	Young generation	Mesozoic generation	Sig.
Mass communication channels	4.22 ± 0.93	4.25 ± 0.89	0.64
Interpersonal channels	3.53 ± 0.97	3.66 ± 1.05	0.05
Organizational channels	3.37 ± 1.11	3.62 ± 1.12	0.001
Social media channels	4.28 ± 0.84	4.19 ± 0.95	0.13

In addition, the results reveal noteworthy changes in health behaviors between the two generations (*t* (777.95) = −2.08, *p* = 0.004). The behavior score of the Mesozoic (*M* = 4.24; SD = 0.72) is significantly higher than that of the young (*M* = 4.09; SD = 0.79) The average difference in scores is −0.15 (*d* = −0.20). 95% CI = [−0.283, –0.070]. Specifically, as demonstrated in Table 4, the Mesozoic outperformed the young regarding frequent hand washing, avoidance of gathering activities, and use of public chopsticks. This conclusion is opposite to the mockery made at the onset of the pandemic by the younger generation, who complained that their parents and elders were unwilling to take preventative precautions. It also suggests that there are still generational differences in health behaviors during the relative calm after the pandemic.

**Table 4:** An analysis of differences in health behaviors between generations.

	Young generation	Mesozoic generation	Sig.
Wearing masks	4.22 ± 0.93	4.25 ± 0.89	0.46
Frequent hand washing	4.33 ± 0.87	4.53 ± 0.76	0.001
Frequent disinfection	3.86 ± 1.13	3.96 ± 1.06	0.18
Avoiding gathering activities	4.21 ± 0.96	4.38 ± 0.88	0.008
Using public chopsticks	3.77 ± 1.22	4.01 ± 1.14	0.003

### 4.2. The mediating effects of cognitive factors

The first four hypotheses predicted that health behaviors would be positively related to perceived severity, perceived susceptibility, self-efficacy, and response efficacy. These hypotheses were tested using multiple linear regression while controlling for participant gender, education level, area, and regional risk. An F-test of the model, *F* (8, 948) = 36.17, *p* < 0.001, Adjusted *R*^2^ = 0.247 was significant. See Table 5 for full information. Perceived severity (*β* = 0.12, *p* < 0.001), self-efficacy (*β* = 0.12, *p* < 0.001) and response efficacy (*β* = 0.40, *p* < 0.001) all exhibit substantial beneficial effects on health behaviors, however perceived susceptibility has a negative influence on health behaviors (*β* = −0.07, *p* = 0.039 < 0.05). Thus, hypotheses 2, 3, and 4 are supported, while hypothesis 1 is not. Regarding the control variables, multiple linear regression analysis reveals that gender substantially influences health behaviors (*β* = 0.15, *p* = 0.015 < 0.05). This indicates that women’s health behaviors are superior to men’s. In addition, education level has a marginally significant negative effect on health behaviors (*β* = −0.05, *p* = 0.072), meaning that the health behaviors performance of the public with a higher education background is not as excellent as that of the public with a lower education background. Lastly, the data indicates that regional risk and whether a location is urban or rural have no meaningful effect on health behaviors.

**Table 5:** Results of multivariate linear analysis for perceived susceptibility, perceived severity, self-efficacy and response efficacy on preventative health behaviors.

Variables	*β*	SE	*t*	*p*	Adjusted *R*^2^
Regional risk	−0.09	0.07	−1.35	0.176	
Area	0.01	0.07	0.10	0.921	
Education level	−0.05	0.03	−1.80	0.072	
Gender	0.15	0.06	2.45	0.015	
Perceived susceptibility	−0.07	0.03	−2.06	0.039	
Perceived severity	0.12	0.03	3.59	<0.001	
Self-efficacy	0.12	0.03	3.71	<0.001	
Response efficacy	0.40	0.03	11.75	<0.001	0.247

The figures are standard coefficients.

Furthermore, bootstrapped confidence intervals (at the 0.05 level with 1,000 re-samples) were examined to check the indirect effect. If the confidence interval does not contain zero, it indicates a significant indirect effect. Indirect effects of media exposure and health behavior accounted for 47%. The results showed that media exposure indirectly influenced health behaviors by perceived severity (H6; 95% CI [0.002, 0.028]), self-efficacy (H7; 95% CI [0.01, 0.03]), response efficacy (H8; 95% CI [0.08, 0.14]), but not by perceived susceptibility (H5). In addition, the path represented by H8 has the most substantial mediation impact. Thus, through increasing response efficacy, media exposure can better improve the health behaviors of individuals (Table 6).

**Table 6:** Testing the mediation effect of media exposure on health behavior through response efficacy, self-efficacy, perceived severity, and perceived susceptibility.

	Effect	BootSE	BootLLCI	BootULCI	Relative effect ratio
TOTAL	0.14	0.02	0.11	0.18	47%
A1	0.11	0.02	0.08	0.14	37%
A2	0.02	0.01	0.01	0.03	7%
A3	0.01	0.01	0.002	0.028	2%
A4	0.001	0.002	−0.002	0.005	
(C1 = A1–A2)	0.09	0.02	0.06	0.13	
(C1 = A1–A3)	0.09	0.02	0.06	0.12	

The figures are standard coefficients; A1 = response efficacy; A2 = self-efficacy; A3 = perceived severity; A4 = perceived susceptibility.

### 4.3. The moderating effect of generation

Model 58 in PROCESS was utilized to evaluate the moderated mediation model to answer RQ3. The findings revealed that generation moderated both of the routes represented by H8. The results are shown in Table 7. Interaction of media exposure and generation predicts perceived susceptibility, *β* = −0.10, SE = 0.05, *p* = 0.05. Interaction between perceived susceptibility and generation predicts health behaviors considerably, *β* = −0.13, SE = 0.06, *p* < 0.05. The mediated conditional effect on perceived susceptibility was negative for the Mesozoic generation, *β* = −0.09, SE = 0.04, 95%CI [−0.17, −0.004], whereas it was not significant for the young generation. Additionally, the conditional effect of perceived susceptibility on health behavior was negative for the Mesozoic generation, *β* = −0.15, SE = 0.05, 95%CI [−0.24, −0.05], but insignificant for the young generation.

**Table 7:** Moderated mediation analysis.

Predictors	Mediator = perceived susceptibility		DV = health behaviors	
	*β*	SE	*β*	SE
Intercept	−0.05	0.11	−0.02	0.09
Response efficacy	−0.18^***^	0.04	0.32^***^	0.03
Self-efficacy	0.0001	0.03	0.10^***^	0.03
Perceived severity	0.38^***^	0.03	0.11^***^	0.03
Media exposure	0.11	0.08	0.15^***^	0.03
Generation	0.03	0.07	0.07	0.06
Media exposure * Generation	−0.10^*^	0.05	–	–
Perceived susceptibility	–	–	0.12	0.09
Perceived susceptibility * Generation	–	–	−0.13^*^	0.06
Direct and indirect effects	Coefficient	95% CI	Coefficient	95% CI
Conditional effects				
Young generation	0.01	−0.06, 0.08	−0.01	−0.09, 0.06
Mesozoic generation	−0.09^*^	−0.17, −0.004	−0.15^**^	−0.24, −0.05
Conditional indirect effects				
Young generation			−0.0002	−0.003, 0.003
Mesozoic generation			0.01	0.0009, 0.0284
Index of moderated mediation			0.01	0.0005, 0.0286

Coefficients presented are standardized estimates. ^*^p < 0.05, ^**^p < 0.01, ^***^p < 0.001.

Moreover, the indices of moderated mediation differed from zero for health behaviors (coefficients were 0.01). This indicated that the indirect effects of media exposure through perceived susceptibility on health behaviors differed for young and Mesozoic generations. For the Mesozoic generation, the indirect effect of media exposure on health behavior was positive, coefficient = 0.01, SE = 0.01, 95%CI [0.0009, 0.0284]. However, For the young generation, the indirect effect of media exposure on health behavior was insignificant. The above reflects the differential effect of the applicability of protection motivation theory among generations in public health events.

## 5. Conclusion and discussion

Firstly, for RQ1 and RQ2 provided in this study, the independent sample *t*-test results revealed that the young and Mesozoic had substantial generational differences in media exposure extensity and health behaviors. After the virus is controlled, the young generation’s media attention swiftly moves to other issues, while the Mesozoic continues to pay close attention to pandemic-related information. In addition, the Mesozoic generation is more likely to get the relevant information through organizational and interpersonal channels. Therefore, they are more likely to use knowledge about the pandemic in dinner conversations. In addition, research has demonstrated that interpersonal pathways are crucial for encouraging beneficial behavior changes (Yang and Chao, 2021). As a result of the Mesozoic’s heightened media attention, their health behaviors were superior to those of the young. As wearing a mask is a mandatory code of conduct in many public places, there is no evident age difference in this regard. However, there are significant generational differences in avoiding gathering activities, washing hands and disinfection, and using public chopsticks. In addition, how can it be explained that young people will complain online about their parents and elders not adopting the appropriate preventive measures in the early stages of the pandemic, when the virus is very contagious, in contrast to the study’s findings? On the one hand, generational differences in health behaviors may show up in a variety of ways depending on the stage of the outbreak. However, as was already indicated, research has shown that during the pandemic, young people expressed their thoughts on contentious subjects more frequently online. The Mesozoic generation may suffer from widespread aphasia, and conclusions drawn from Internet public opinion may not be accurate reflections of reality.

Then, based on social cognitive theory and protection motivation theory, this research examines the impact of social-cognitive predictors on health behaviors and then develops a mediating model of media exposure on health behaviors. Multiple regression analysis indicated that perceived severity (H2), self-efficacy (H3), and response efficacy (H4) all had favorable effects on health behaviors, but contrary to expectations, perceived susceptibility (H1) had an adverse impact on health behaviors. And media exposure can influence health behaviors *via* the mediating effects of perceived severity (H6), self-efficacy (H7), and response efficacy (H8), but not *via* perceived susceptibility (H5), of which the path (H8) is the most influential. This indicates that media exposure is most effective at influencing health behaviors by improving their response efficacy during flattening periods. In addition, for RQ3, moderated mediation analysis revealed that generation moderates the indirect influence of media exposure on health behaviors *via* perceived susceptibility. Media exposure had a positive indirect effect on the health behavior of the Mesozoic generation. However, this indirect effect was not substantial for the young population. Specifically, media exposure adversely affected the Mesozoic’s perceived susceptibility, which has a negative effect on their health behaviors. Thus, media exposure positively affects Mesozoic health behaviors by reducing their perceived susceptibility.

At the time of the study, the pandemic in China had reached a plateau, and except for sporadic confirmed cases, most areas had been cleared of confirmed cases. Therefore, the media is no longer a source of risk information but instead emphasizes the pandemic’s positive status and prevention knowledge. During this period, the Mesozoic continues to pay close attention to information on the pandemic, thereby decreasing their susceptibility. What ‘s more, the relationship between risk perception and health behaviors is complex. Previous studies have inconsistent conclusions on the effects of perceived susceptibility and perceived severity on health behaviors. Both negative and positive associations were found in the studies investigating the relationship between risk perceptions and behaviors. Through meta-analysis, the researchers believe that a considerable part of these inconsistent conclusions is methodological issues, such as replacing behavior with behavior intentions, not setting risk conditions, etc. (Brewer et al., 2007). Some experts have also indicated that optimism bias influences respondents’ evaluations of health threats (Weinstein, 1987). The optimism bias may lessen the perceived need to avoid unfavorable outcomes by engaging in healthy practices (Larsman et al., 2012). Druică et al. (2020) define optimism bias as the tendency to estimate a lower likelihood of experiencing negative health events than others. COVID-19 is a pandemic with a long incubation period, highly contagious, and highly dependent on crowd interaction for transmission, making all members of society universally susceptible compared to other health topics such as skin cancer (Yi et al., 2020). However, the perceived susceptibility measure in the study centered on the evaluation of oneself being infected with the virus, and the findings revealed a low perceived susceptibility (*M* = 1.84; SD = 1.01). And new research has confirmed the optimism bias of the COVID-19 instance in China (Today.uconn.edu, 2020). In light of this, it is feasible to speculate that there is an optimism bias in the public’s perception of the current risk. Both Druică et al. (2020) and Chowdhury et al. (2014) found that optimism bias increases with age. Hence, the effect of susceptibility on health behaviors may be more strongly adversely moderated by optimism bias in the Mesozoic generation, reducing their intention to engage in health behaviors.

In addition, a multiple regression study revealed that education level has a marginally significant negative influence on health behaviors. And the variables’ correlation analysis (shown in Table 2) revealed that education level was adversely associated with perceived severity. This indicates that those with higher levels of education are more hopeful about the pandemic situation in the country, hence decreasing their willingness to engage in health activities. A comparable study (Zhang et al., 2020) also revealed that persons with relatively low levels of education exhibited increased anxiety and concern for themselves during the pandemic. This may have a convincing explanation according to the extended parallel process model. In line with protective motivation theory, the extended parallel process model validates the positive impacts of perceived severity, perceived susceptibility, self-efficacy, and response efficacy on health behaviors (Witte and Allen, 2000). Nonetheless, it claims that individuals do not develop additional cognitive or affective responses when the perceived threat is low and that people’s preventative behaviors are only driven when they perceive a sufficient level of threat in response to risk information.

Furthermore, the implication of this study is that the development of health communication theory must take into account the different effects of generations and the disease-specific characteristics. Future study can build and enhance the existing theory by testing the applicability of each variable in specific situations. And when the current pandemic enters a lull and the Chinese government continues to stress the importance of maintaining healthy preventative behaviors, effective health communication is crucial. In this sense, we propose that positive information about the pandemic should be shared alongside risk information in order to heighten the public’s risk perception and encourage preventive behaviors. Moreover, in future health communication, due to systematic differences between generations in terms of external environmental exposure and cognitive-psychological factors, risk communication should focus on refined and precise services, and differentiated measures should be taken for various groups in terms of media channels and media content. For instance, to persuade the Mesozoic generation, the interpersonal and organizational channels should be emphasized, whereas, for the young generation, government officials can use social media to widely disseminate information about the pandemic and adopt novel content forms to attract their attention. Since optimism bias is one of the primary barriers to engaging in risk reduction practices, future risk communication programs should explore ways to correct this misunderstanding.

In conclusion, the main limitations of this article are reflected in: First, because of the use of cross-sectional data, the causal effect relationship cannot be adequately tested. Meanwhile, the actual behavior data cannot be obtained through self-assessment questionnaire. The behavior measurement in the questionnaire actually measures attitudes and concepts toward health behaviors. Additionally, due to the limitations of online surveys, it was impossible to pay attention to the old generation, that is, the public above 55, and only compared the Mesozoic and the young generation. As marginalized groups utilizing new media, their media exposure and health behaviors in public health events deserve consideration; Lastly, generation is a topic worthy of consideration, and explaining generational differences in health behavior due to media exposure is only one perspective. To attempt a fuller understanding of generational difference in health behavior, it will be important to do additional qualitative research on topics such as generational differences in culture. Some researchers suggest cultural differences may exist in the association between risk perception and health behavior (Lau et al., 2010).

## Data availability statement

The raw data supporting the conclusions of this article will be made available by the authors, without undue reservation.

## Author contributions

RH contributed to the conception, data analysis, and manuscript writing of the study. JH contributed to parts of the conception, data analysis, and manuscript writing of the study. HZ contributed to the literature obtaining and analysis, manuscript writing, and performed the analysis with constructive discussions of the study. All authors contributed to the article and approved the submitted version.

## Funding

This work was supported by National Social Science Foundation Art Project: Research on Creative Transformation and Innovative Development of Traditional Culture in Jiangnan. Project Approval Number: 20BH153.

## Conflict of interest

The authors declare that the research was conducted in the absence of any commercial or financial relationships that could be construed as a potential conflict of interest.

## Publisher’s note

All claims expressed in this article are solely those of the authors and do not necessarily represent those of their affiliated organizations, or those of the publisher, the editors and the reviewers. Any product that may be evaluated in this article, or claim that may be made by its manufacturer, is not guaranteed or endorsed by the publisher.

## Footnotes

^1^ https://wj.qq.com
